# Supplementary figures and images for: Circum-Mediterranean influence in the Y-chromosome lineages associated with prostate cancer in Mexican men: A Converso heritage founder effect?
Source: PLoS One. 2024 Aug 16;19(8):e0308092. doi: 10.1371/journal.pone.0308092 (PMC11329122; doi:10.1371/journal.pone.0308092)

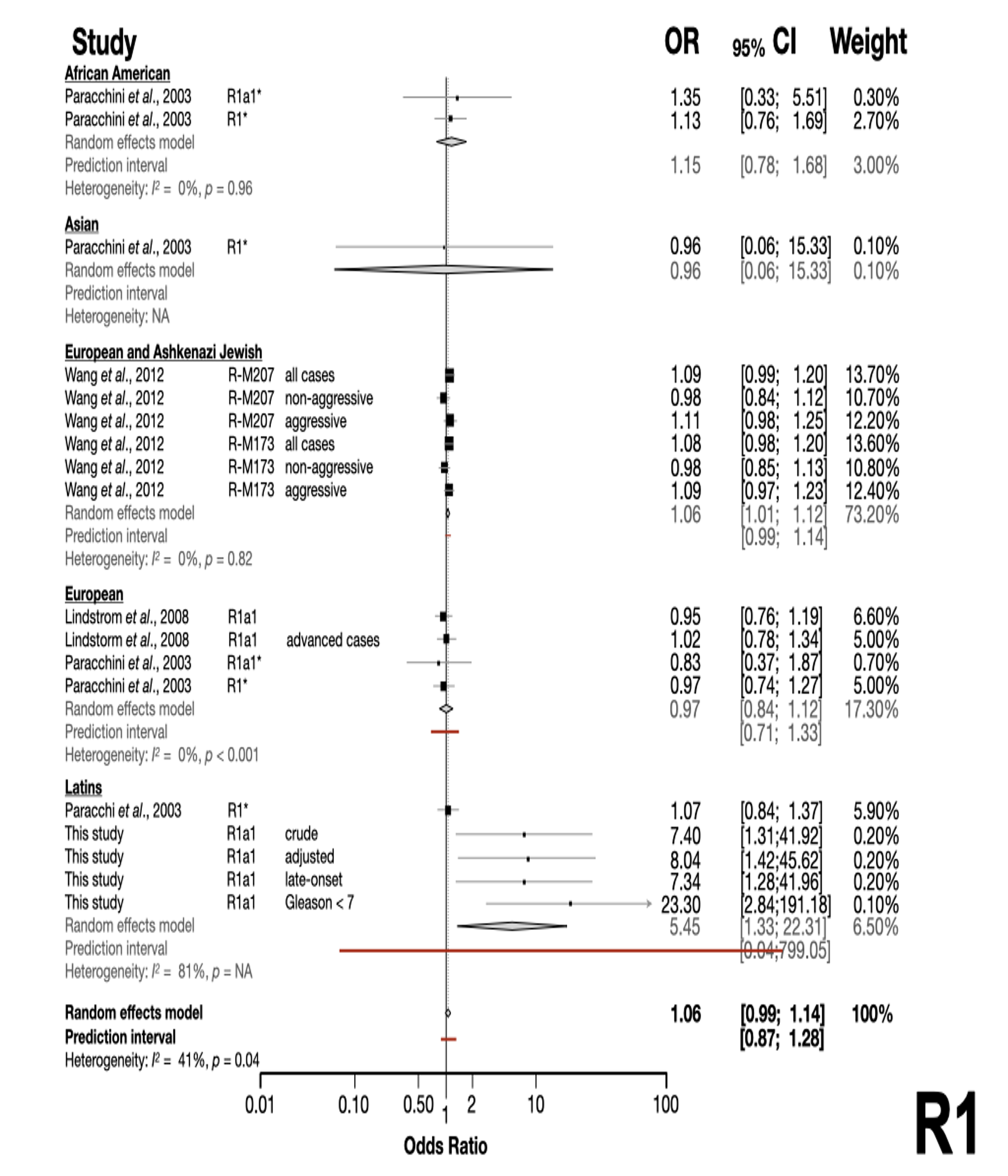

Supplement: S1 Fig — OR, odds ratio; CI, confident intervals; I2, proportion of the variance (heterogeneity). All data were obtained from several published studies. (TIF) [file pone.0308092.s001.tif]

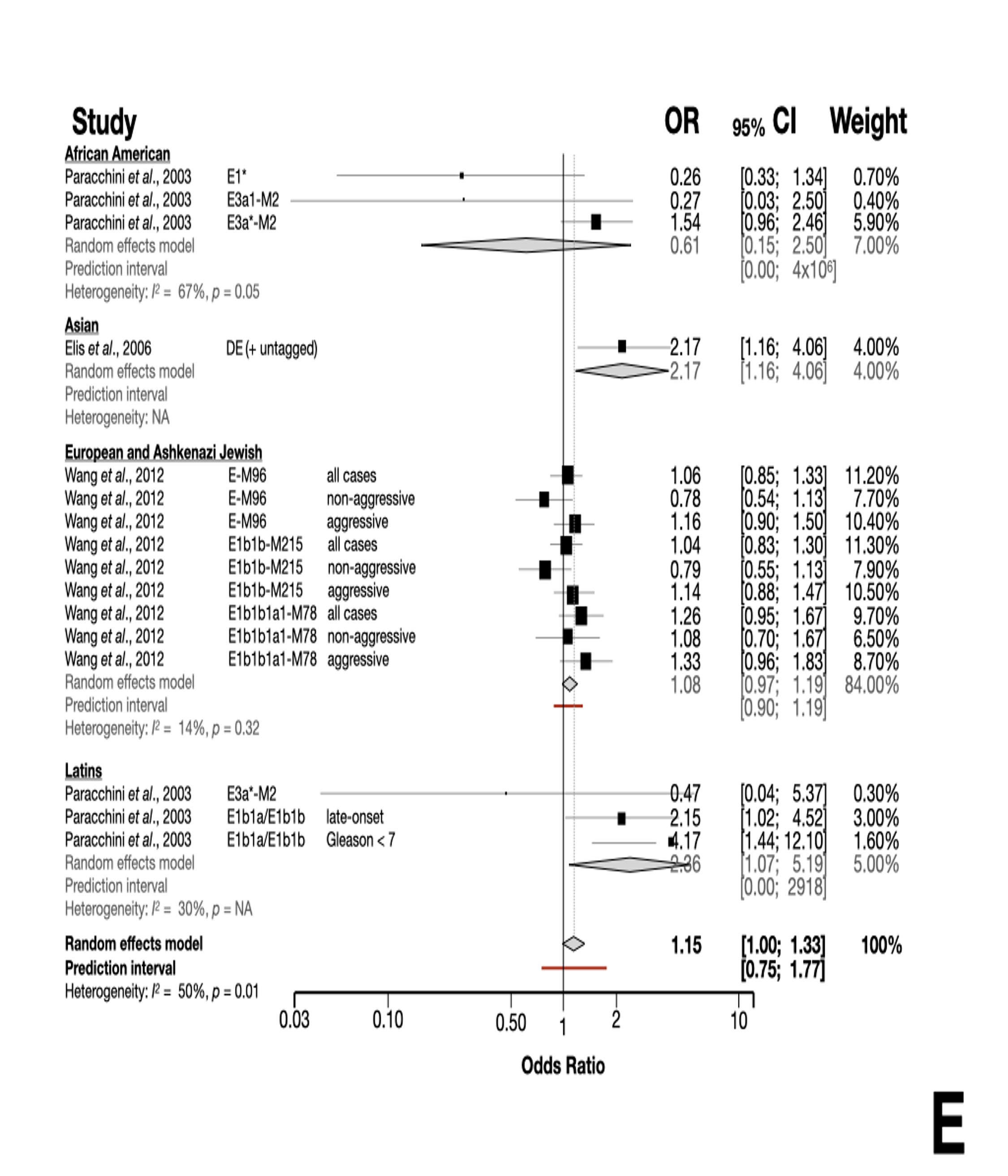

Supplement: S2 Fig — OR, odds ratio; CI, confident intervals; I2, proportion of the variance (heterogeneity). All data were obtained from several published studies. (TIF) [file pone.0308092.s002.tif]

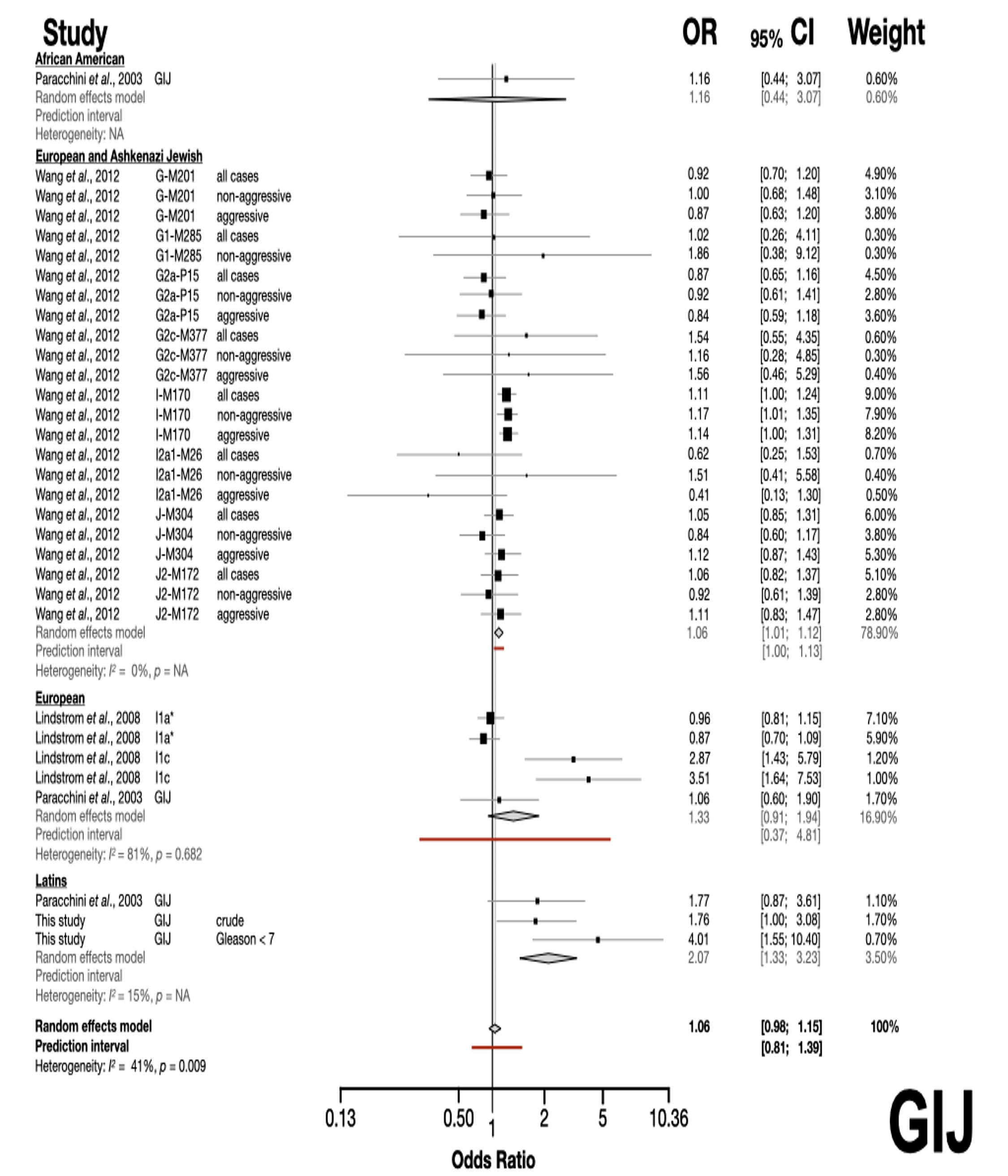

Supplement: S3 Fig — OR, odds ratio; CI, confident intervals; I2, proportion of the variance (heterogeneity). All data were obtained from several published studies. (TIF) [file pone.0308092.s003.tif]

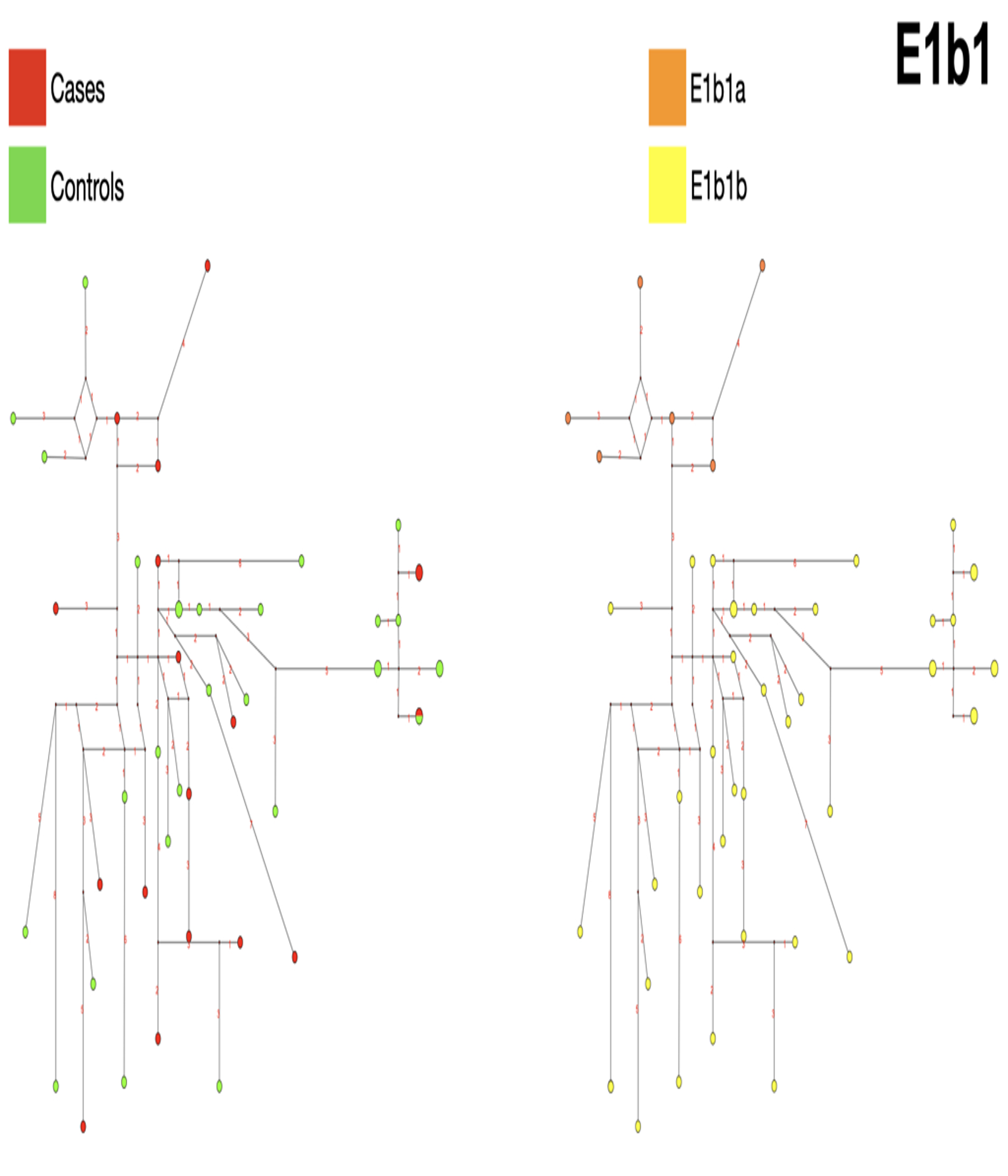

Supplement: S4 Fig — Numbers in red represent the number of differences between one haplotype and other(s). (TIF) [file pone.0308092.s004.tif]

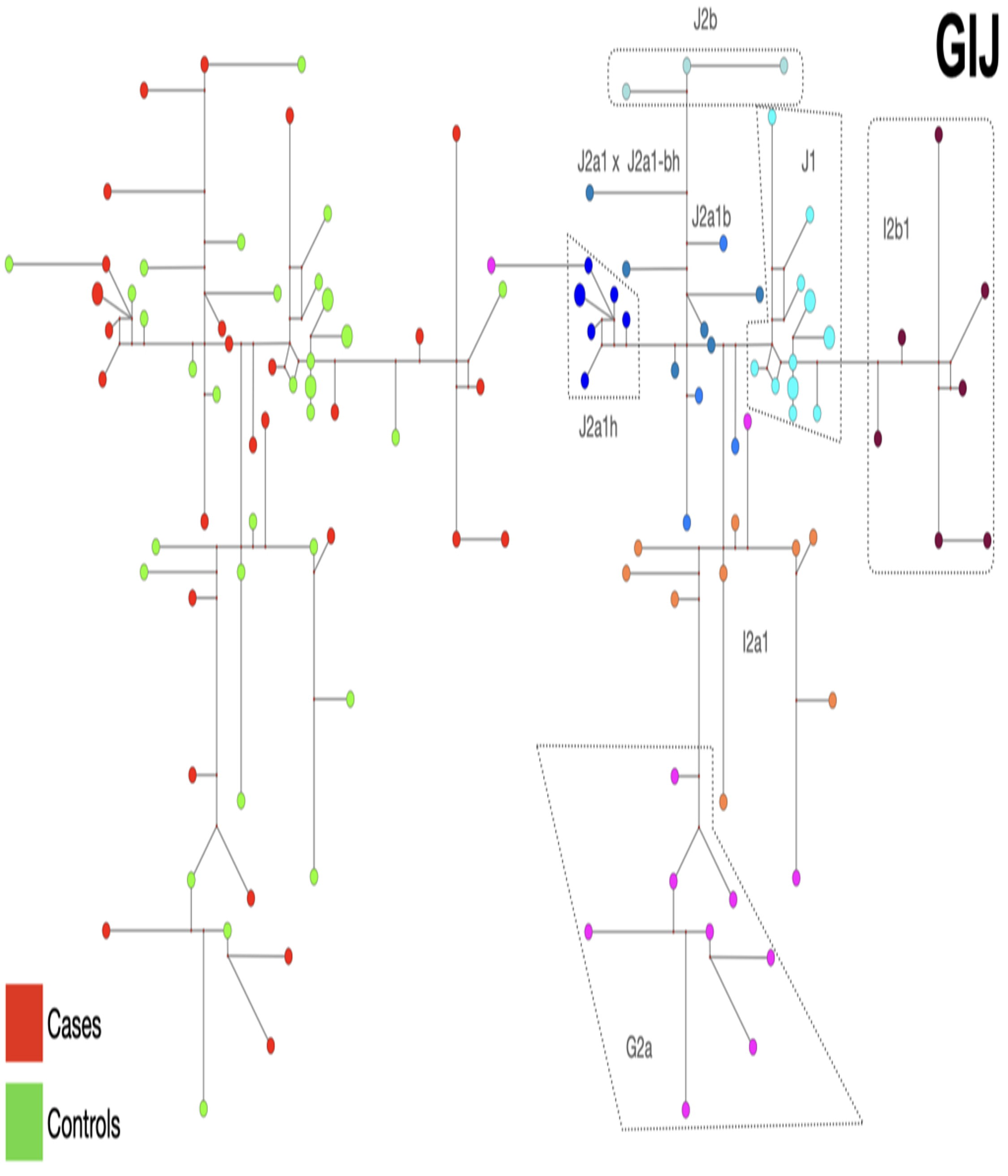

Supplement: S5 Fig — Numbers in red represent the number of differences between one haplotype and other(s). (TIF) [file pone.0308092.s005.tif]

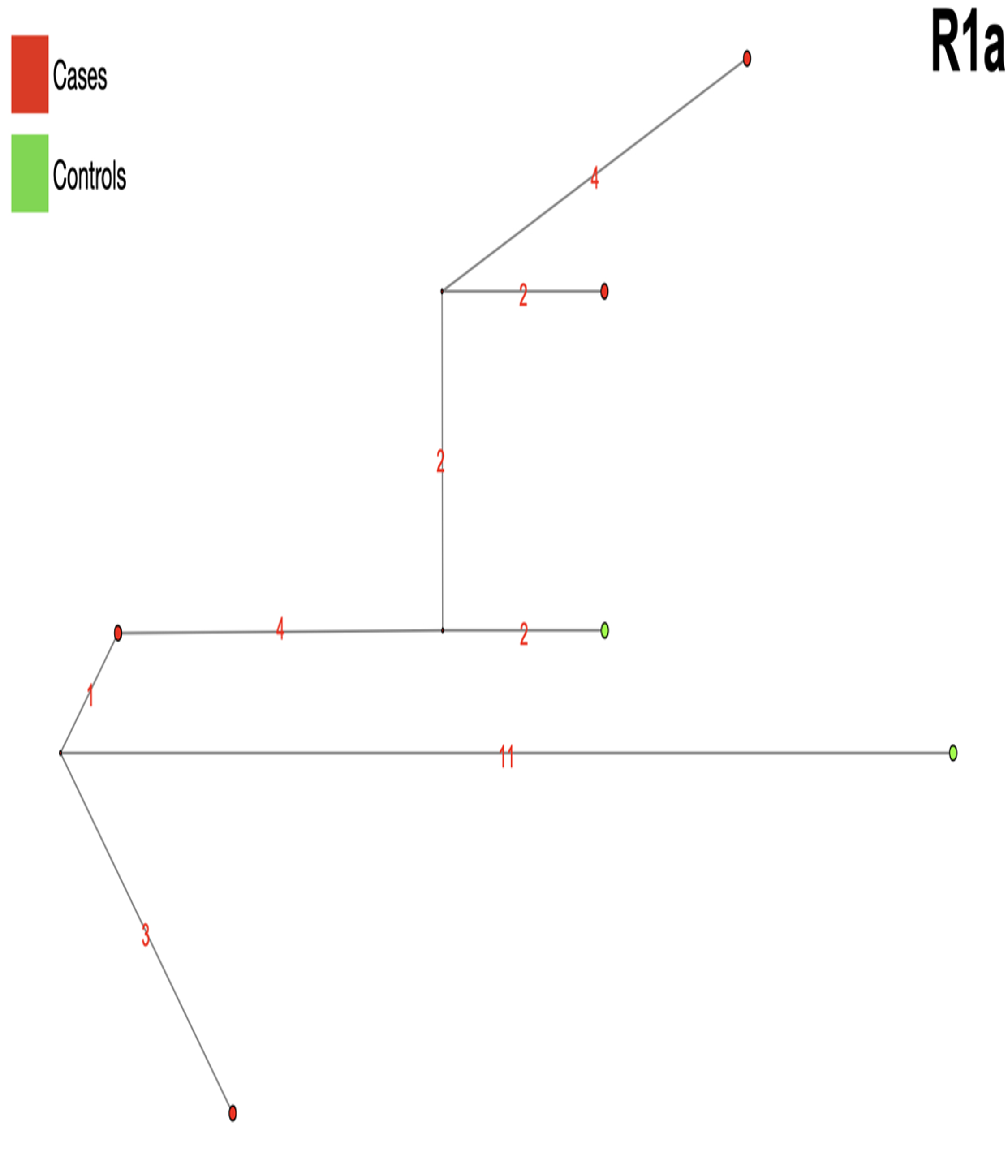

Supplement: S6 Fig — Numbers in red represent the number of differences between one haplotype and other(s). (TIF) [file pone.0308092.s006.tif]

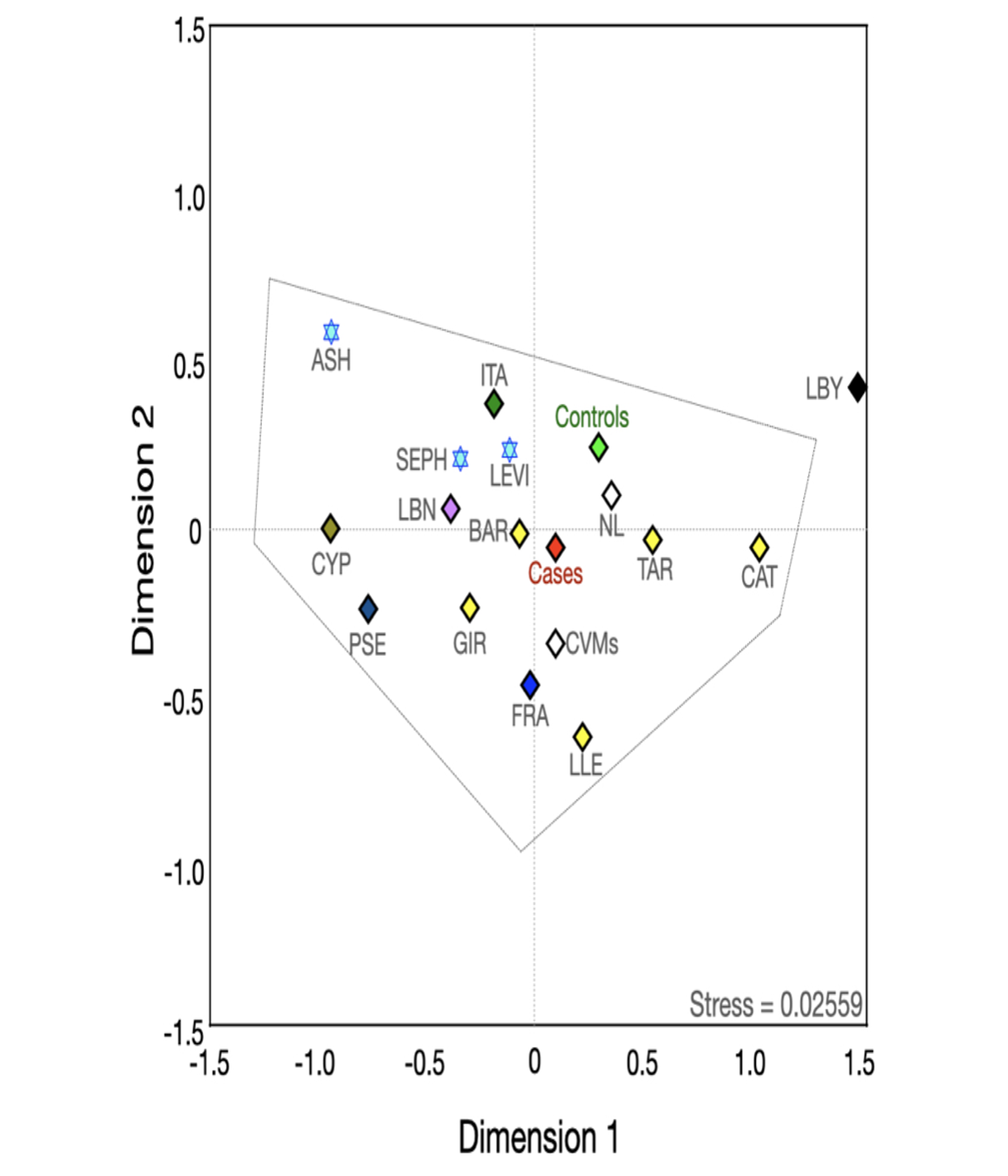

Supplement: S7 Fig — ASH, Ashkenazi Jews; BAR, Barcelona; CAT, Cataluña; CVMs (Central Valley of Mexico, data from Santana et al); CYP, Cyprus; FRA, France; GIR, Girona; ITA, Italy; LBN, Lebanon; LBY, Libya; LEVI, Levites Jews; LLE, Lleida; NL, Nuevo Leon; PSE, Palestinian Territory; SEPH, Sephardic Jews; TAR, Tarragona. All p values were adjusted with the method of false discovery rates. The dotted lines indicate that no significant differences were found among the populations. (TIF) [file pone.0308092.s007.tif]

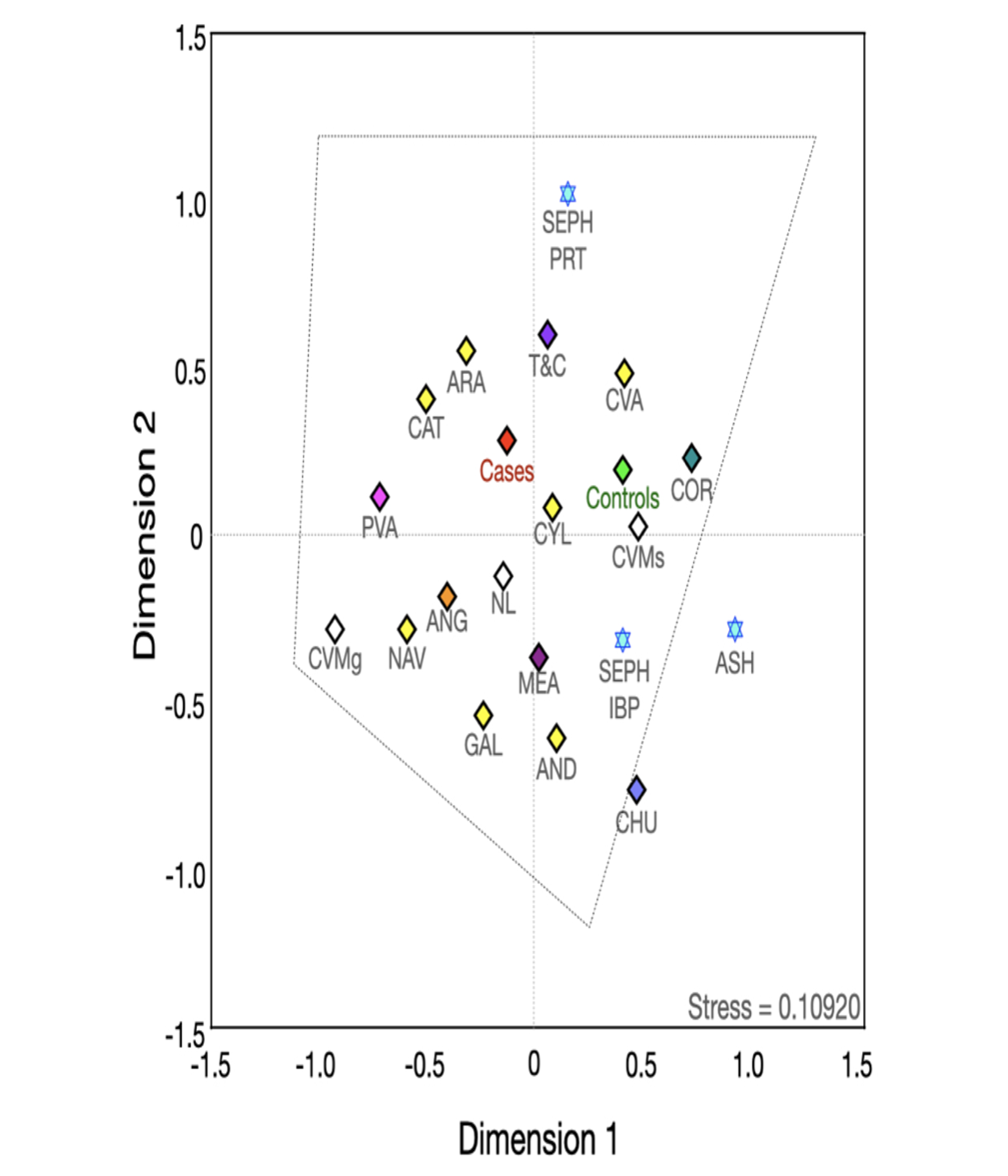

Supplement: S8 Fig — AND, Andalusia; ANG, Anatolian Greeks; ARA, Aragon; ASH, Ashkenazi Jews; CAT, Cataluña; CHU; Chuetas; COR, Corsica; CVA; Valencian Community; CVMg (Central Valley of Mexico, data from Gomez et al); CVMs (Central Valley of Mexico, data from Santana et al); CYL, Castilla and Leon; GAL, Galicia; MEA, Middle East; NAV, Navarra; NL, Nuevo Leon; PVA, Basque Country; SEPH-IBP, Sephardic Jews from the Iberian Peninsula; SEPH-PRT, Sephardic Jews from Portugal; T&C, Turkish and Cypriots. All p values were adjusted with the method of false discovery rates. The dotted lines indicate that no significant differences were found among the populations. (TIF) [file pone.0308092.s008.tif]

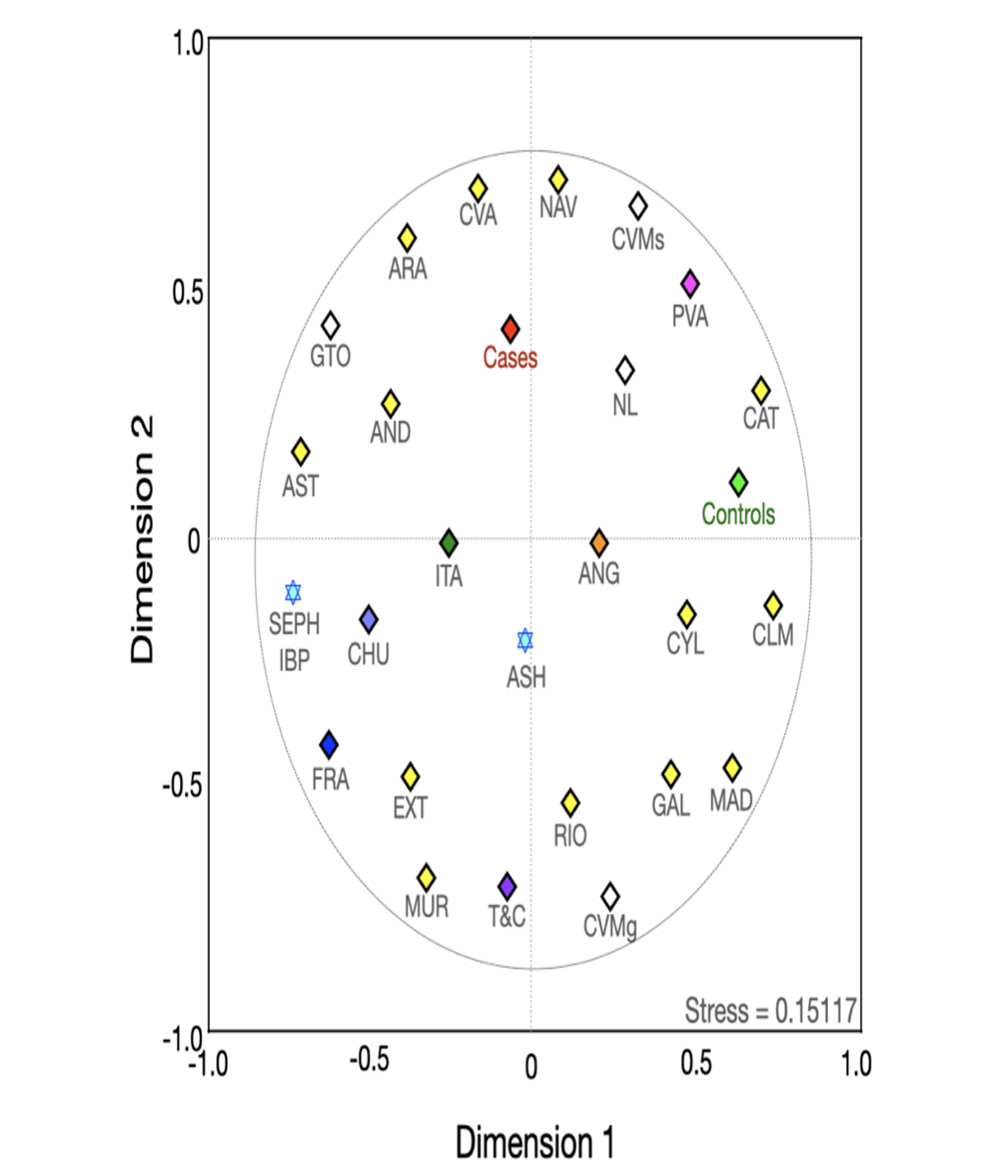

Supplement: S9 Fig — AND, Andalusia; ANG, Anatolian Greeks; ARA, Aragon; ASH, Ashkenazi Jews; AST, Asturian Community; CAT, Cataluña; CHU; Chuetas; CLM, Castilla la Mancha; CVA; Valencian Community; CVMg (Central Valley of Mexico, data from Gomez et al); CVMs (Central Valley of Mexico, data from Santana et al); CYL, Castilla and Leon; EXT, Extremadura; FRA, France; GAL, Galicia; GTO, Guanajuato; ITA, Italy; MAD, Madrid; MEX, Mexico; MUR, Murcia; NAV, Navarra; NL, Nuevo Leon; PVA, Basque Country; RIO, La Rioja; SEPH-IBP, Sephardic Jews from the Iberian Peninsula; T&C, Turkish and Cypriots. All p values were adjusted with the method of false discovery rates. The dotted lines indicate that no significant differences were found among the populations. (TIF) [file pone.0308092.s009.tif]

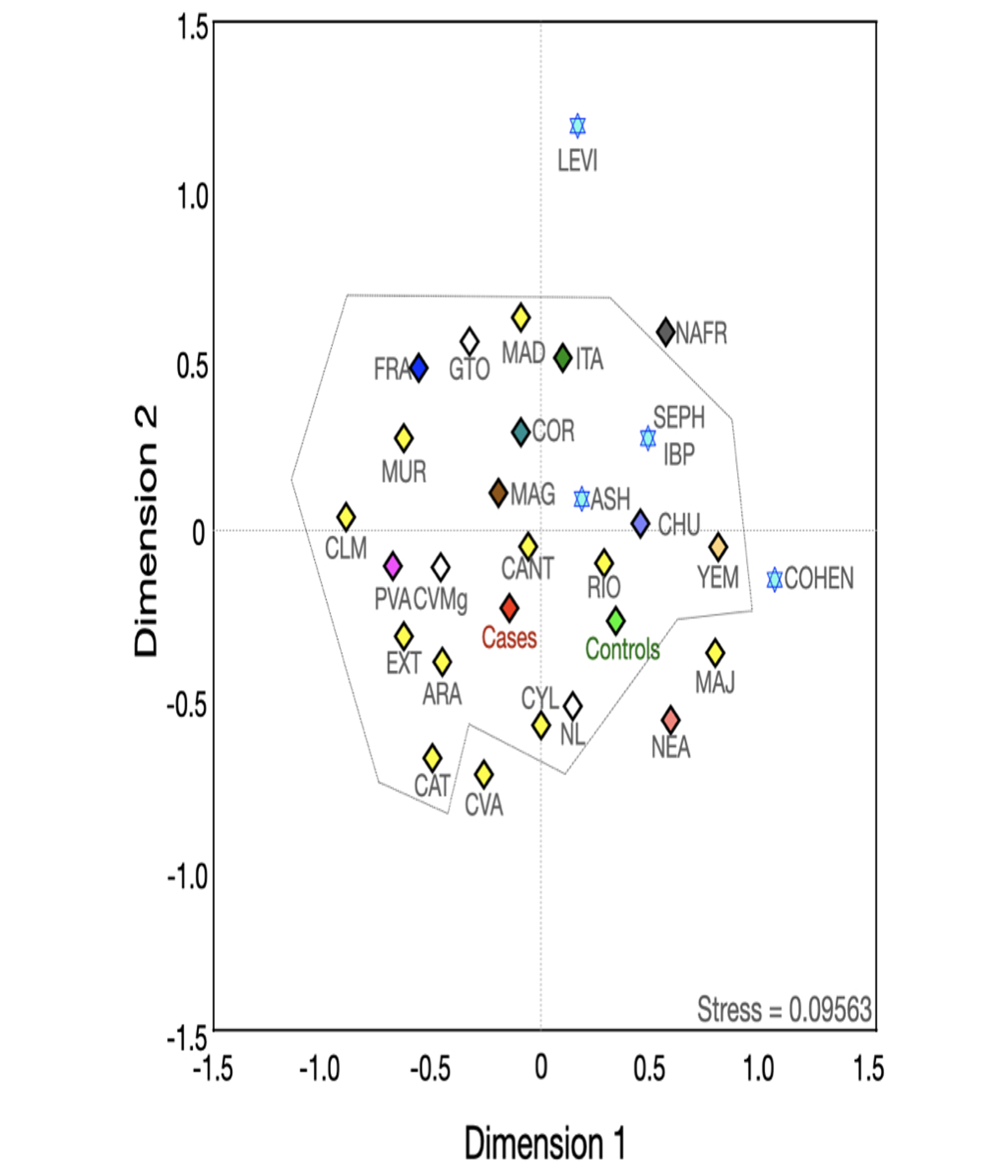

Supplement: S10 Fig — ARA, Aragon; ASH, Ashkenazi Jews; CANT, Cantabria; CAT, Cataluña; CHU; Chuetas; CLM, Castilla la Mancha; COHEN, Cohen Jews; COR; Corsica; CVA; Valencian Community; CVMg (Central Valley of Mexico, data from Gomez et al); CYL, Castilla and Leon; EXT, Extremadura; FRA, France; GTO, Guanajuato; ITA, Italy; LEVI, Levites Jews; MAD, Madrid; MAG, Maghreb; MAJ, Majorca; MUR, Murcia; NAFR, North Africa; NEA, Near East; NL, Nuevo Leon; PVA, Basque Country; RIO, La Rioja; SEPH-IBP, Sephardic Jews from the Iberian Peninsula; YEM, Yemen. All p values were adjusted with the method of false discovery rates. The dotted lines indicate that no significant differences were found among the populations. (TIF) [file pone.0308092.s010.tif]

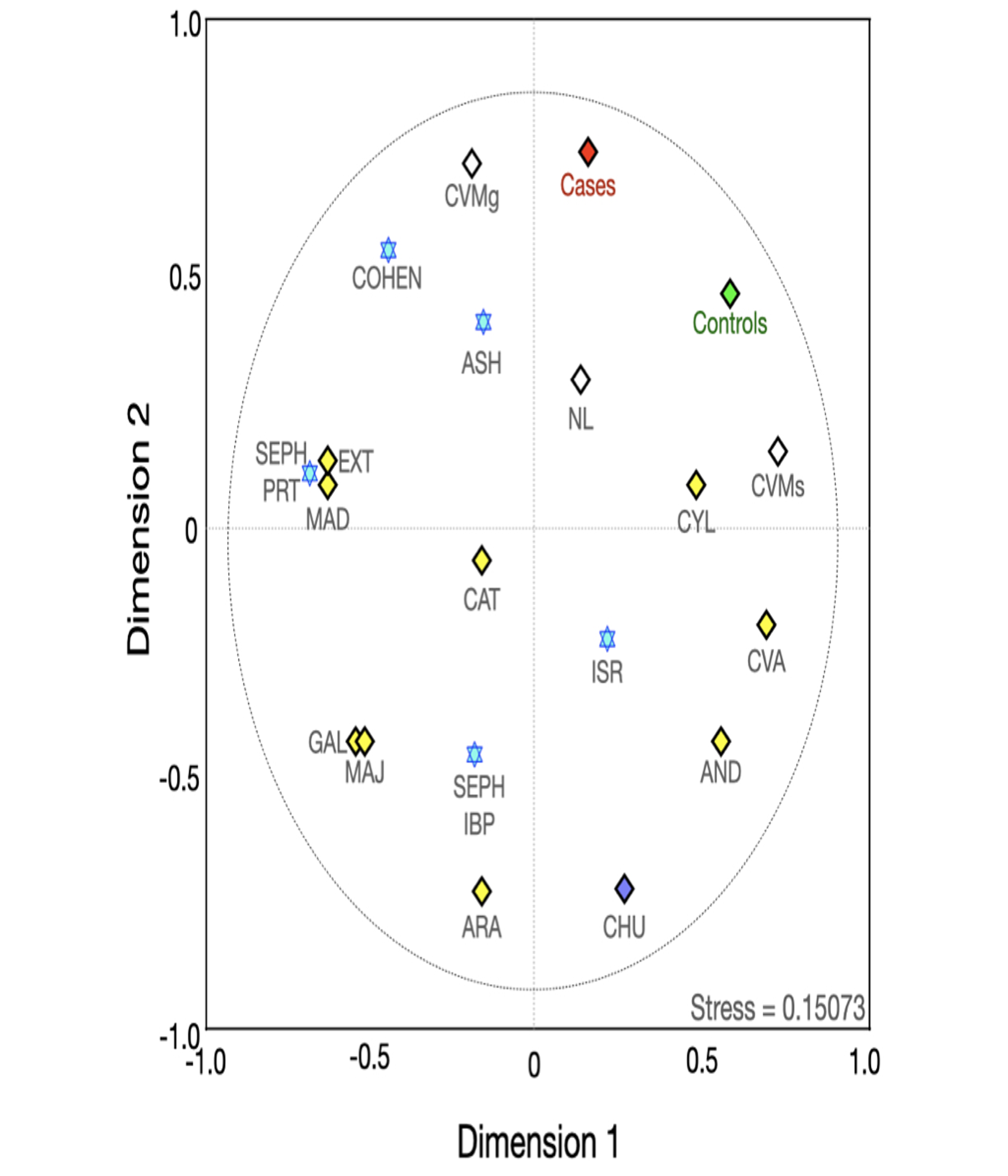

Supplement: S11 Fig — AND, Andalusia; ARA, Aragon; ASH, Ashkenazi Jews; CAT, Cataluña; CHU; Chuetas; COHEN, Cohen Jews; CVA; Valencian Community; CVMg (Central Valley of Mexico, data from Gomez et al); CVMs (Central Valley of Mexico, data from Santana et al); CYL, Castilla and Leon; EXT, Extremadura; GAL, Galicia; ISR, Jews from Israel; MAD, Madrid; MAJ, Majorca; NL, Nuevo Leon; SEPH-IBP, Sephardic Jews from the Iberian Peninsula; SEPH-PRT, Sephardic Jews from Portugal. All p values were adjusted with the method of false discovery rates. The dotted lines indicate that no significant differences were found among the populations. (TIF) [file pone.0308092.s011.tif]

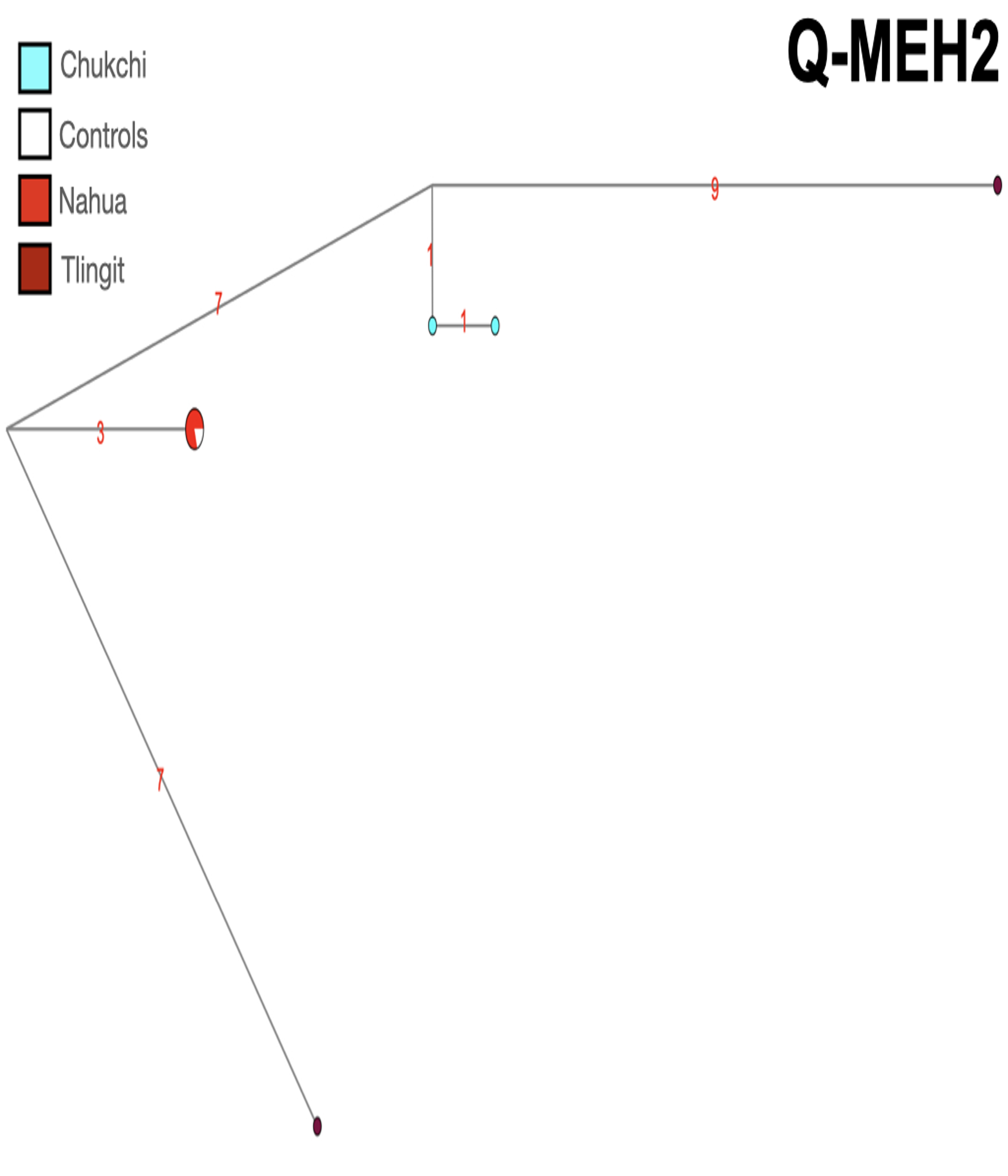

Supplement: S12 Fig — Numbers in red represent the number of differences between one haplotype and other(s). (TIF) [file pone.0308092.s012.tif]

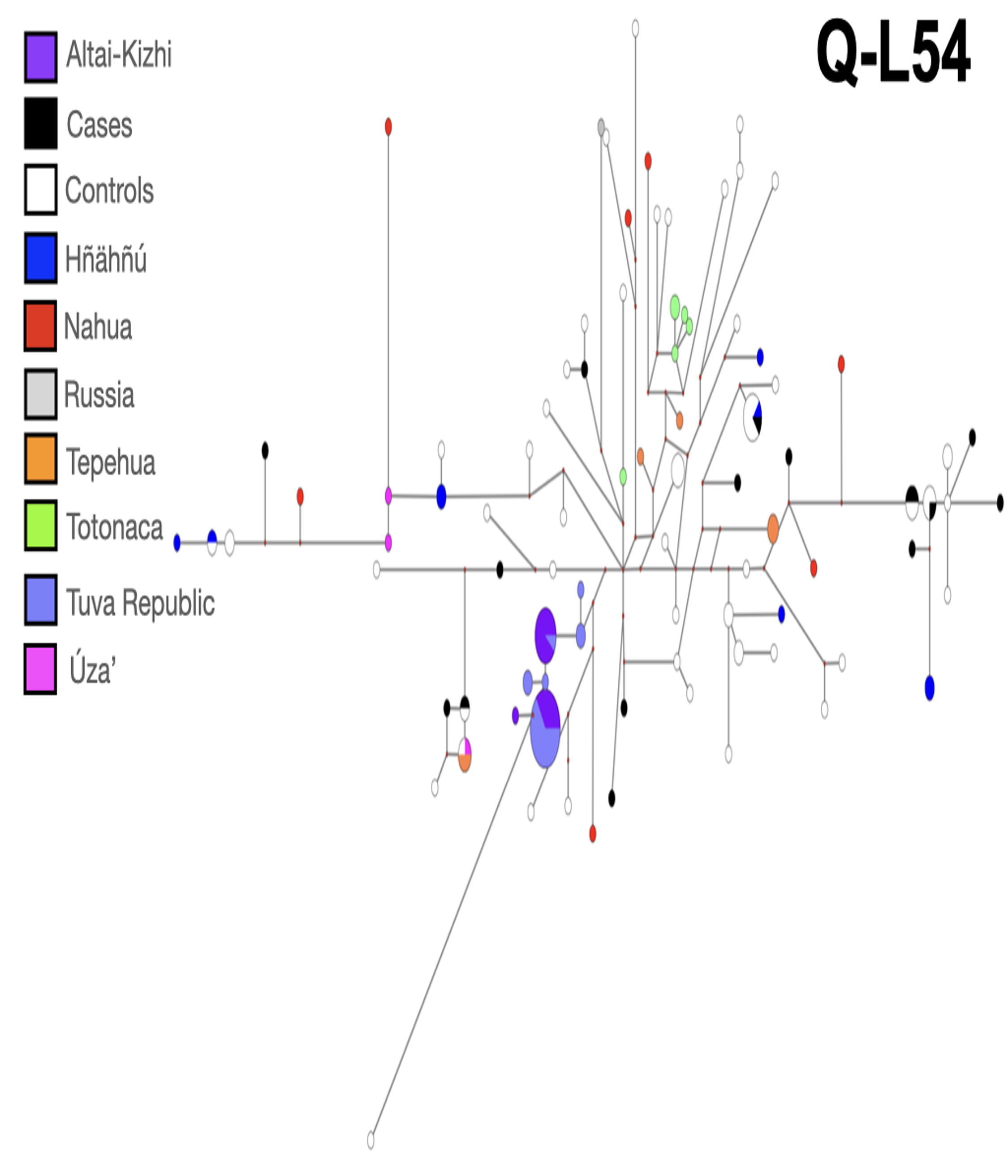

Supplement: S13 Fig — (TIF) [file pone.0308092.s013.tif]

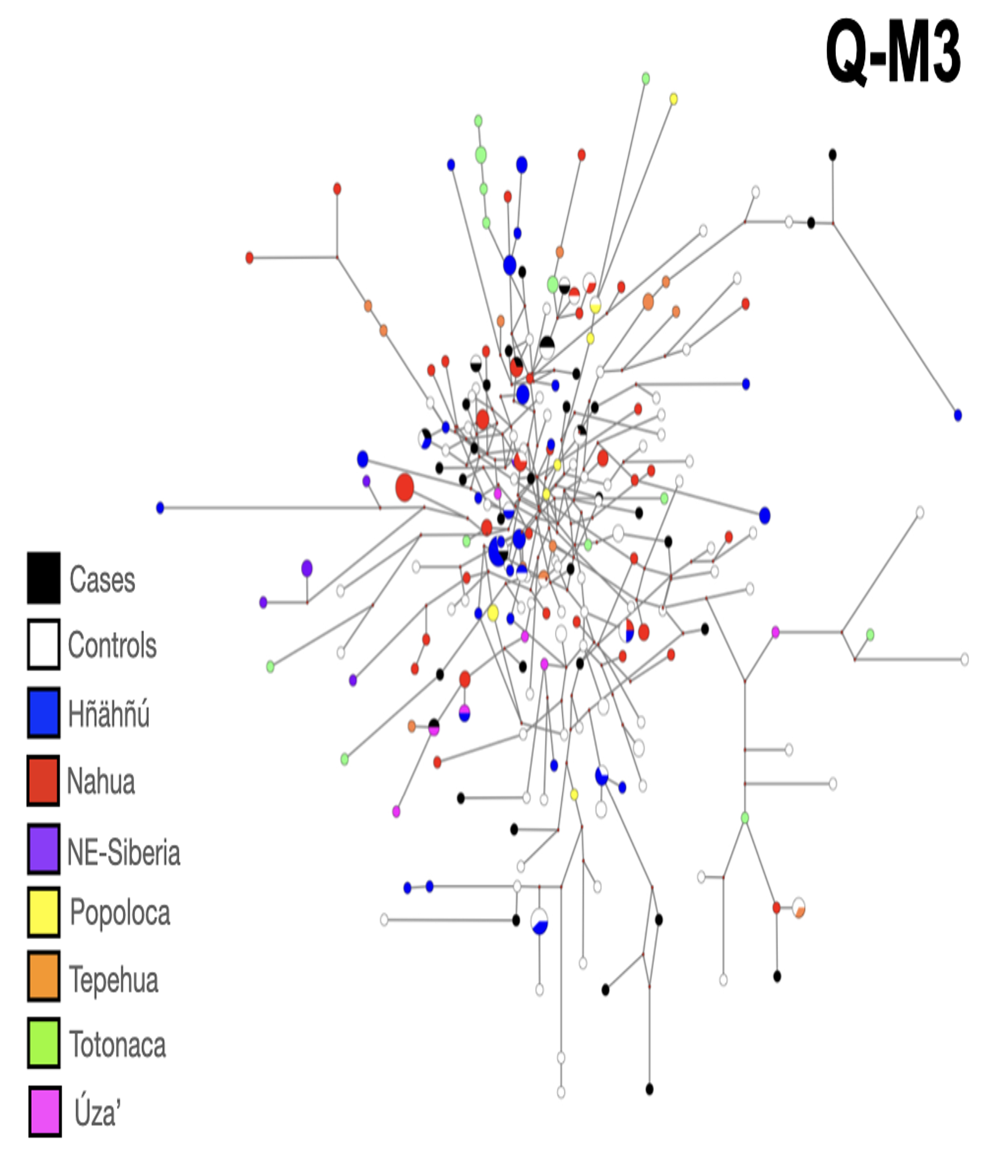

Supplement: S14 Fig — (TIF) [file pone.0308092.s014.tif]
